# The association between hyperuricemia and coronary artery calcification development: A systematic review and meta‐analysis

**DOI:** 10.1002/clc.23266

**Published:** 2019-09-30

**Authors:** Ling Liang, Xianghua Hou, Kevin R. Bainey, Yanlin Zhang, Wayne Tymchak, Zhongquan Qi, Weihua Li, Hoan Linh Banh

**Affiliations:** ^1^ Department of Cardiology The First Affiliated Hospital of Xiamen University Xiamen China; ^2^ Department of Cardiology First Clinical Medical College of Fujian Medical University Fuzhou China; ^3^ Department of Nephrology The First Affiliated Hospital of Xiamen University Xiamen China; ^4^ Department of Nephrology First Clinical Medical College of Fujian Medical University Fuzhou China; ^5^ Division of Cardiology, Mazankowski Alberta Heart Institute University of Alberta Edmonton Alberta Canada; ^6^ Institute of Organ Transplantation Xiamen University Xiamen China; ^7^ Department of Family Medicine University of Alberta Edmonton Alberta Canada

**Keywords:** coronary artery calcification, hyperuricemia, meta‐analysis

## Abstract

Hyperuricemia coincides with coronary artery calcification (CAC) development, but the role of serum uric acid (SUA) as a risk factor for CAC remains unclear. The objective of this study was to gain an insight into the association between SUA and CAC in adults by performing a meta‐analysis. MEDLINE, EMBASE, the Cochrane Library, and EBSCO (CINAHL) were searched for relevant observational studies published until 2 June 2019. Studies were included only if they reported data on CAC presence (Agatston score > 0) or progression related to hyperuricemia in subclinical adult patients. The pooled estimates of crude and adjusted odds ratios (ORs) and 95% confidence interval (CI) were calculated to evaluate the association between CAC presence or progression and hyperuricemia. A total of 11 studies were identified involving 11 108 adults. The pooled OR based on the frequency of CAC presence showed that patients in the high SUA group had 1.806‐fold risk for developing CAC (95% CI: 1.491‐2.186) under the minimal threshold of hyperuricemia (more than 6 mg/dL or 357 μmoL/L). When SUA levels were analyzed as categorical variables, the pooled estimate of adjusted ORs was 1.48 (95% CI: 1.23‐1.79) for CAC presence. Additionally, for each increase of 1 mg/dL of SUA level, the risk of CAC progression was increased by 31% (95% CI: 1.15‐1.49) with an average follow‐up duration ranged from 4.6 to 6.1 years. Hyperuricemia is closely associated with increased risk of CAC development and CAC progression in asymptomatic patients.

## 1 INTRODUCTION

Coronary artery calcification (CAC) is a marker of coronary atherosclerosis^1^ and is associated with major adverse cardiovascular events. CAC can be measured by chest computerized tomography (CT) and quantified by the Agatston Score.^2^ This method is a validated gauge of atherosclerotic plaque burden and able to provide noninvasive quantitative information of all coronary artery vessels. Its presence and progression correlate not only with the development and extent of coronary heart disease (CHD),^3^ but also with CHD mortality and all‐cause mortality.^3^, ^4^

Hyperuricemia may increase cardiovascular risk^5^ by inducing endothelial dysfunction,^6^ oxidation stress,^7^ and inflammation.^8^ Studies have showed that increased serum uric acid (SUA) level is associated with adverse clinical events and mortality with acute coronary syndromes^9^, ^10^ as well as stable ischemic heart disease.^11^ However, a few Mendelian randomization studies^8^, ^12^, ^13^ have demonstrated inconsistent results examining the causal relationship of increased serum urate concentration and CHD. Similarly, the role of SUA as a risk factor for CAC remains controversial as some studies have reported a significant association between SUA and CAC,^14^, ^15^, ^16^, ^17^, ^18^ while others suggest no significant association.^19^, ^20^ Given these inconsistencies, the primary objective of this systematic review is to assess the association between SUA and CAC in adult patients.

## 2 METHOD

The current systematic review was performed in accordance with the checklist of meta‐analysis of observational studies in epidemiology.^21^ A review protocol was not mandated as part of the systematic review.

### 2.1 Search strategy

We performed a comprehensive literature search for relevant studies evaluating the association between hyperuricemia and CAC from four major electronic databases: MEDLINE, EMBASE, the Cochrane Library, and EBSCO (CINAHL), using the following heading MeSH terms and keywords: [uric acid OR hyperuricemia OR urate] AND [Coronary artery calcification OR coronary calcification OR coronary artery calcium score OR coronary artery calcium scoring OR Coronary calcium OR Coronary calcium score OR Coronary calcium scoring OR coronary artery calcinosis OR coronary calcinosis OR calcification of Coronary artery OR coronary artery calcium]. The search included all studies published up to 2 June 2019, with no language restriction. The studies were manually screened. A full electronic search strategy (no limits) performed in MEDLINE can be reviewed in the Supporting Information Appendix.

### 2.2 Study eligibility

The study inclusion criteria were: (a) adult subjects; (b) describing the association between hyperuricemia and CAC; (c) the definition of CAC presence determined as the Agatston score over 0^22^; (d) CAC progression defined as participants whose square root‐transformed CAC volume (calcium volume scores) increase by ≥2.5 mm^23^; (e) CAC reported as the primary outcome, unadjusted, or adjusted odds ratios (ORs) with 95% confidential interval estimates for CAC presence and CAC progression; (f) patients without CAD or CHD or CKD or gout; and (g) patients not receiving treatment for hyperuricemia. No geographic restriction was applied.

### 2.3 Study selection

Two reviewers (LL and XHH) independently screened the titles and abstracts to determine the inclusion of the studies. Full texts of the selected studies were read to further screen for eligible studies. Abstracts from conference or meetings were used to find the related published articles. Attempts were made to contact the original authors for additional details if necessary. Any discrepancy was resolved by a third reviewer (HLB) to reach a consensus.

### 2.4 Data abstraction and quality assessment

Two reviewers (LL and XHH) independently extracted all data by using a standardized data abstraction excel file to retrieve information about studies features (first authors, publication years, publishing journals, and study types), participants information (gender, geographical location, sample size, and basic diseases), cutoff levels for hyperuricemia, outcome definition, confounders, duration of follow‐up, the frequency of CAC presence, and ORs. The primary outcome was the risk estimate for the association between hyperuricemia and CAC. The secondary outcome was the risk for CAC progression. Given only observational studies were found, the Newcastle‐Ottawa Scale was applied for quality assessment^24^ based on three components as follows: selection of the study groups (0‐5 points for cross‐sectional study, 0‐4 points for cohort study and case‐control study), comparability of study groups (0‐2 points), and ascertainment of the interest outcome (0‐3points). The score ranges from 0 to 10 points, with a higher score indicating better methodological quality. Discrepancies were resolved by third reviewer (HLB) and fourth reviewer (ZYL).

### 2.5 Statistical analysis

The conventional unit (milligram per deciliter) was used for all SUA levels. A conversion rate of 0.01681 (1 μmoL/L = 0.01681 mg/dL) was used to standardize all SUA levels. The cutoff values for hyperuricemia differed among studies (Table 1).

**Table 1:** Summary of the studies

First author	Year	Journal	Age (year)	Sample size (%men)	Participants	Hyperuricemia definition (mg/dL)	Confounding factors	Outcome definition	Type of study	NOS score	Follow‐up duration
Raul D. Santos	2007	*American Journal of Cardiology*	48 ± 7	371 (100)	Brazil man, white, nondiabetic subjects free of known CHD	≥7.1	Age, SBP, waist circumference, HDL‐C, TG, glucose, smoking, physical activity, and WBC count, MetS	CAC score > 0	Cross‐section	9 (4//3)	—
Ticiana C. Rodrigues	2010	*Diabetes Care*	38.5 ± 8.3	969 (46)	United States, individuals asymptomatic for CAD	Per 1 mg/dL increase	Age, gender, type 1 diabetes, baseline CVS, HTN, smoking, HDL‐C, LDL‐C	Progression of CAC	Retrospective cohort	9 (4/2/3)	6.0 ± 0.5 years
Eswar Krishnan	2011	*Arthritis Research and Therapy*	40 ± 4	2498 (48)	US young adults free of CKD, diabetes from CARDIA trial	M: > 6.7,F: > 4.7	Age, gender, race, HDL‐C, LDL‐C,TG, smoking, BP class, MetS, CRP, waist circumference, alcohol use, creatinine, and serum albumin	CAC score > 0	Cross‐section	9 (4/2/3)	—
Cao Hui‐li	2013	*Chinese Journal of Epidemiology*	60.3 ± 11.02	903 (48)	China, natural population in Beijing	≥7.1	Gender, age, BMI, creatinine, hsCRP, SBP, DBP, FPG, TC, TG, HDL—C, smoking, alcohol use	CAC score > 0	Cross‐section	9 (4/2/3)	—
Aslı İnci Atar	2013	*The Anatolian Journal of Cardiology*	53.6 ± 10.5	442 (77)	Turkey, suspected CHD with a low‐intermediate risk for CAD	>5.6 per 1 mg/dL increase	Age, smoking and 10‐year total risk of Framingham risk score	CAC score > 0	Case control	8 (4/2/2)	—
Chagai Grossman	2014	*The Journal of Clinical Hypertension*	55.5 ± 7.3	663 (85)	Israel, men above 40 and women above 50, free of CVD	>6.1	Age, gender, HTN, eGFR, BMI, diabetes, hyperlipidemia	CAC score > 0	Prospective cross‐section	9 (4/2/3)	—
Petter Bjornstad	2014	*Acta Diabetologica*	36.5 ± 9	652 (46)	United States, asymptomatic for CVD, with or without type 1 diabetes	Per 1 mg/dL increase	Age, diabetes duration, HbA1c, HDL‐C, SBP, DBP, and antihypertensive medications	CAC progression, CAC score > 0	Prospective cohort	9 (4/2/3)	Average 6.1 years
Richard Y. Calvo	2014	*American Journal of Cardiology*	62.2 ± 6.4	368 (0)	United States, Filipino women, and Non‐Hispanic, white women	Per 1 mg/dL increase	Age, follow‐up time, HTN, diabetes,﻿ statin use and visceral adiposity, ﻿estrogen use	CAC progression, CAC score > 0	Retrospective cohort	9 (4/2/3)	Average 4.6 years
Rehan Malik	2016	*Aging Clinical and Experimental Research*	84.5 ± 4.2	208 (21)	Brazilian octogenarians (C80 years) free from known clinical CVD	—	Gender, BMI, SBP, DBP, antihypertensive treatment, diabetes, use of oral hypoglycemic agents, TC, HDL‐C, LDL‐C, TG, and creatinine clearance	CAC score > 0	Prospective cross‐section	9 (4/2/3)	—
Loretta Zsuzsa Kiss	2018	*Journal of Cardiovascular Translational Research*	60 ± 10.9	281 (41)	Hungarian healthy adults	—	Gender, BMI, Diabetes, age, smoking, creatinine, HTN, hyperlipidemia	CAC score > 0	Cross‐section	9 (4/2/3)	—
Paulo H. Harada	2019	*Journal of Cardiology*	49 (44‐55)	3753 (46)	Brazilian, Sao Paulo site participants of the ELSA‐Brasil cohort	—	Age, gender, race/ethnicity, family history of CAD, alcohol use, smoking, physical activity, waist circumference, diabetes, HTN, HDL‐C, TG, hsCRP	CAC score > 0	Cross‐section	9 (4/2/3)	—

Abbreviations: BMI, body mass index; CAC, coronary artery calcification; CAD, coronary artery disease; CHD, coronary heart disease; CKD, chronic kidney disease; CRP, C reactive protein; CVD, cardiovascular disease; CVS, calcium volume scores; DBP, diastolic blood pressure; eGFR, estimated glomerular filtration rate; FPG, fasting plasma glucose; HDL‐C, high‐density lipoprotein cholesterol; hsCRP, high sensitivity C reactive protein; HTN, hypertension; LDL‐C, low‐density lipoprotein cholesterol; MetS, metabolic syndrome; SBP, systolic blood pressure; TC, total cholesterol; TG, triglyceride; WBC, white blood cell.

Based on the frequency of CAC presence (CAC score > 0) (among both hyperuricemia and normouricemia groups) for each study, the pooled estimates of ORs and 95% confidence intervals (CIs) were calculated to evaluate the crude relationship between SUA and CAC.

As for the confounders, adjusted ORs in each study were combined to evaluate the association of SUA as a categorical variable for CAC prevalence. The prediction of SUA on CAC progression was performed by pooling adjusted ORs.

We evaluated the presence of heterogeneity across trials by using the *I* ^2^ statistic. If *I* ^2^ is <50% and *P* value is >.1, heterogeneity is acceptable. If *I* ^2^ is >50% and *P* value is <.1, we would adopt random effect or a meta‐regression method to find sources for the obvious heterogeneity. To assess the potential publication bias, we conducted the visually symmetric funnel plot and quantified Egger test.

A two‐tailed *P* value <.05 was considered statistically significant. All the statistical analyses were performed in Stata 15.1 (Stata Corp, College Station, Texas).

## 3 RESULTS

### 3.1 Search and selection of studies

**Figure 1:**
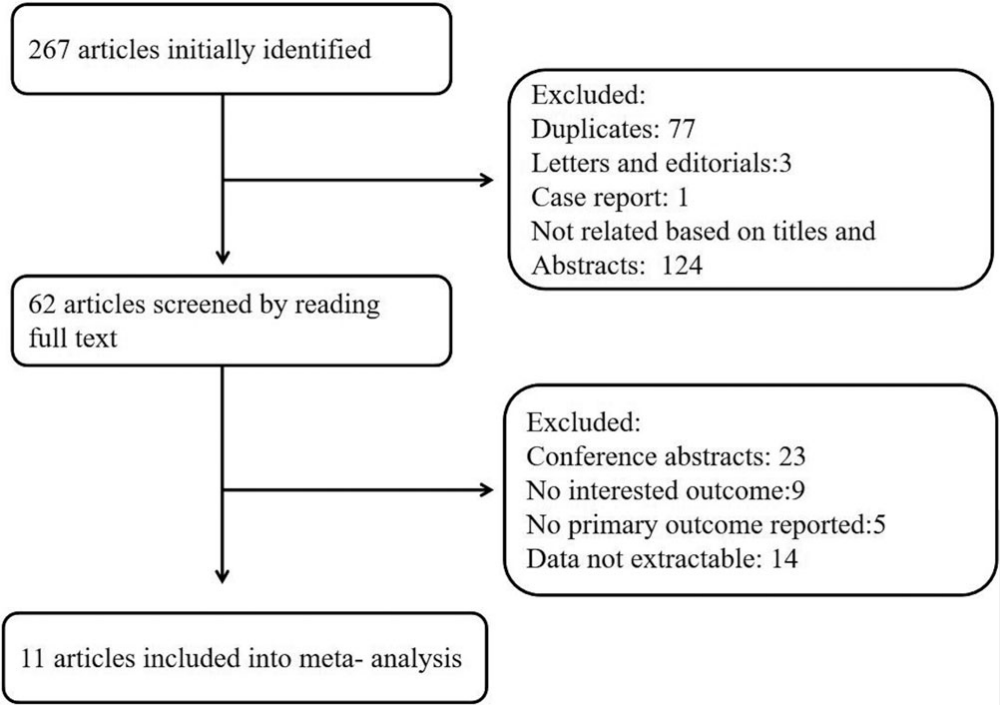
Flow diagram for the search process

The initial electronic database search identified 267 articles and there were 77 duplicates. A total of 124 irrelevant articles were excluded after screening by titles and abstracts. One case report and three letters were excluded. After reading the remaining 62 articles in full text, 11 studies^14^, ^15^, ^17^, ^18^, ^19^, ^25^, ^26^, ^27^, ^28^, ^29^, ^30^ were included totally (Figure 1). There were 11 108 participants included in the meta‐analysis.

### 3.2 Included studies

The characteristics of the included studies and their participants are summarized in Table 1. Of the 11 included studies (all observational), four were conducted in the United States,^17^, ^18^, ^25^, ^27^ one in Europe,^15^ three in Asia,^14^, ^28^, ^30^ and three in Latin America.^19^, ^26^, ^29^ All studies were published in recent 12 years (2007‐2019) in English except one (Chinese).^14^ The sample size of the studies ranged from 208^19^ to 3753^29^ participants. The average duration of follow‐up was between 4.6 years^17^ and 6.1 years.^18^ The average age of participants ranged from 36.5^18^ to 84.5.^19^ There are one case control,^30^ three cohorts,^17^, ^18^, ^25^ and seven cross‐sectional studies^14^, ^15^, ^19^, ^26^, ^27^, ^28^, ^29^ in total. Of these studies, nine included both genders,^14^, ^15^, ^18^, ^19^, ^25^, ^27^, ^28^, ^29^, ^30^ one included only men,^26^ and one included only women.^17^ The definition of hyperuricemia cutoff value ranged from 5.6 to 7.1 mg/dL in men and from 4.7 to 7.1 mg/dL in women. There were seven studies^14^, ^15^, ^19^, ^26^, ^27^, ^28^, ^29^ that reported the association between hyperuricemia and CAC based on the SUA category subgroup, three on gender subgroup^18^, ^25^, ^27^ and one on race subgroup.^17^ Three cohort studies^17^, ^18^, ^25^ reported the association between hyperuricemia and CAC progression. All of the selected studies were assessed as high quality according to the NOS scale (10 studies^14^, ^15^, ^17^, ^18^, ^19^, ^25^, ^26^, ^27^, ^28^, ^29^ have NOS scores as 9 and 1 study^30^ as 8). See Table 1.

### 3.3 Crude association between SUA and CAC incidence

**Figure 2:**
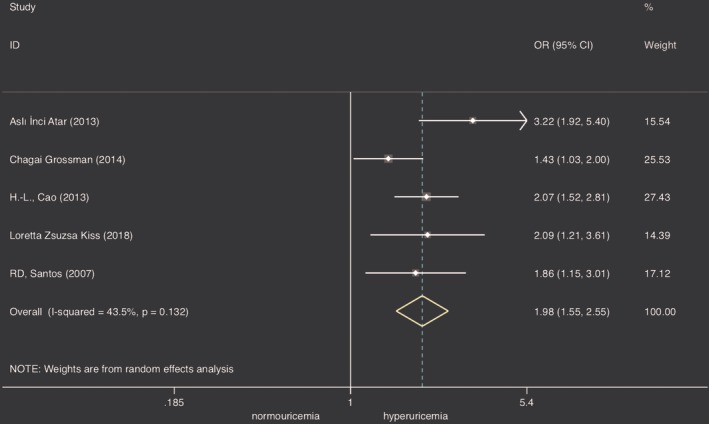
Forest plot of association between hyperuricemia and CAC prevalence after one article deleted. CAC, coronary artery calcification

Five studies^14^, ^15^, ^26^, ^28^, ^30^ were selected to analyze the association between CAC incidence and SUA level. Meta‐analysis showed that patients in the high SUA group had a higher risk of CAC incidence (n = 436, 63%) than patients in the normouricemia group (n = 897, 46%) using a random model (OR: 1.98, 95% CI: 1.55‐2.55). *I* ^2^ was 43.5% (<50%). In order to reduce the heterogeneity, the data with the minimal threshold level of hyperuricemia (more than 6 mg/dL or 357 μmoL/L) were used for further analysis. There was no change in the pooled result (OR: 1.806, 95% CI: 1.491‐2.186) under fixed model with no observed heterogeneity (*I* ^2^ = 0%, *P* = .415) (Figure 2) after one study^30^ was excluded because the authors defined hyperuricemia level as 5.6 mg/dL (333 μmoL/L). The funnel plot was symmetrical and Egger test *P* value was .782, meaning no significant publication bias.

### 3.4 Risk prediction of high SUA on CAC presence

**Figure 3:**
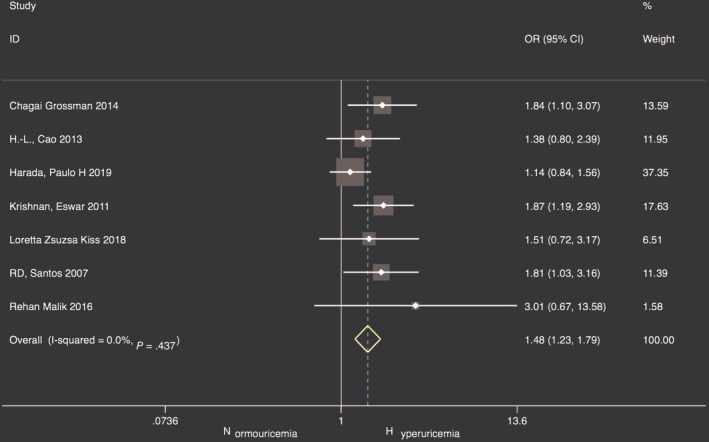
Forest plot of pooled adjusted ORs for CAC presence in the highest SUA category. CAC, coronary artery calcification; ORs, odds ratios

The adjusted ORs extracted from seven studies^14^, ^15^, ^19^, ^26^, ^27^, ^28^, ^29^ for CAC prevalence were analyzed with SUA as a categorical variable. Three studies^15^, ^19^, ^28^ used tertile for SUA stratification and three studies^14^, ^27^, ^29^ used quartile, while only one^26^ study analyzed SUA concentrations strata as dichotomy. In the highest SUA category, the pooled estimated OR was 1.48 (95% CI: 1.23‐1.79) with no observed heterogeneity (*I* ^2^ = 0%, *P* = .437) (Figure 3). The funnel plot was symmetrical and result of Egger test was not statistically significant (*P* = .085) which suggested that there was no serious small studies effect.

### 3.5 Association between SUA and CAC progression

**Figure 4:**
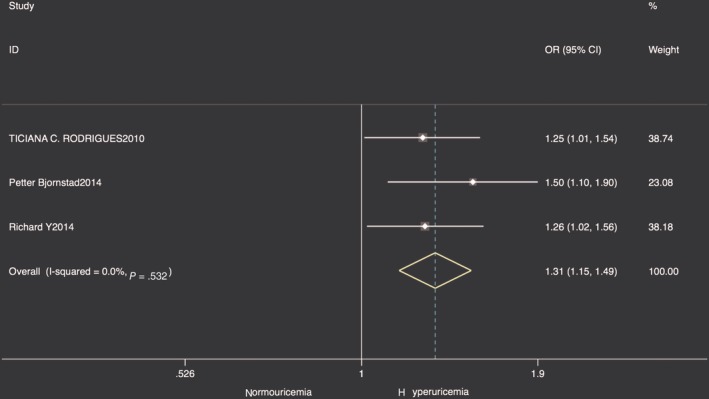
Forest plot of pooled ORs for CAC progression. CAC, coronary artery calcification; ORs, odds ratios

The pooled evaluation of adjusted ORs for CAC progression based on three cohort studies^17^, ^18^, ^25^ was 1.31 (95% CI: 1.15‐1.49) with no observed heterogeneity (Figure 4). The average follow‐up year ranged from 4.6 to 6.1. The funnel plot was asymmetrical and Egger test result showed small size publication bias (*P* = .013).

## 4 DISCUSSION

The results of our meta‐analysis provided a new insight into the association between SUA and CAC development in subclinical patients. We found that the odds of developing CAC were increased by 81% in patients with hyperuricemia. When the highest SUA category was compared with the lowest SUA category, the pooled adjusted estimate showed that the risk of CAC presence was almost 1.5‐fold. Moreover, the risk of CAC progression was increased by up to 1.31‐fold with an average follow‐up duration ranged from 4.6 to 6.1 years.

SUA, a novel risk factor, has been associated with the development of subclinical cardiovascular disease (CVD).^28^ In addition, the risk of mortality and severity of CHD are increased in patients with hyperuricemia.^31^, ^32^ The results from this meta‐analysis support these findings as we have found that SUA is associated with subclinical CAC. There are several possible mechanisms that can explain the association. In vitro, uric acid has stimulated primary vascular smooth muscle cells (VSMC) to produce inflammatory cyclooxygenase‐2 and superoxide anion which contribute to the pathogenesis of atherosclerosis.^33^ In an animal model, high uric acid levels have been shown to cause premature atherosclerosis by disturbing lipid metabolism, promoting the proliferation of VSMCs, and more importantly, activating inflammation.^34^ Additionally, aortic calcification has occurred earlier (more severe) in the high uric acid group compared with the normal diet group and high fat diet group. It is of interest to note the longer exposure to hyperuricemia, the more severe the calcium deposition in the medial layer of blood vessels.^34^ In a middle cerebral artery occlusion rat model conducted by Song and Zhao,^35^ uric acid feeding has led to endothelial cell shed and significant drop of nitric oxide which have initiated and accelerated the atherosclerosis progression.

Since hyperuricemia is a risk factor for early stage of atherosclerosis, therapeutic agents targeting lower uric acid levels would be of interest. Colchicine is widely used, well tolerated, and effective for prevention and treatment of acute gout which is due to hyperuricemia. As uric acid crystals and cholesterol crystals are activated by the same pathway in the pathogenesis of atherosclerosis, colchicine can play a protective role in CVD patients^36^ by rapidly reducing high sensitivity C reactive protein (hs‐CRP),^37^ stabilizing the atherosclerosis plaque,^38^ and reducing cardiovascular events at low dose.^39^ Nevertheless, xanthine oxidase inhibitors, which are another agents used to lower SUA level, have not reduced mortality in patients with CVD.^40^ Future studies are needed to evaluate the efficacy of different types of uric acid lowering drugs on reducing the risk of CAC development and progression.

There were several limitations in this meta‐analysis. First, a strong publication bias was observed with regards to CAC progression in only three cohort papers. The small sample size publication bias and null results unpublished bias may explain this publication bias. Second, the vast majority of selected 11 studies were conducted in developed countries. The results from this meta‐analysis may not be applied to under‐developed countries where different diet and lifestyle would affect the association. Third, although a multivariable adjustment was conducted in most of the included studies, confounding effects from other unadjusted risk factors may exist. Notably, none of the included studies has been adjusted for diet, which significantly influences the SUA level. Despite these limitations, this is the first meta‐analysis to analyze the relationship between hyperuricemia and CAC.

## 5 CONCLUSION

This systematic review and meta‐analysis showed an association between hyperuricemia and increased risk of CAC development and CAC progression in asymptomatic patients. Our findings suggested that patients with hyperuricemia should be monitored closely for coronary atherosclerosis.

## AUTHOR CONTRIBUTIONS

W.H.L. and Z.Q.Q. conceived and conceptualized the research idea. L.L., X.H.H. and H.L.B. performed the screening, full text assessment, quality assessment and data extraction and Z.Q.Q. approved the data. L.L. and X.H.H. did data analyses, Z.Y.L. contributed and Z.Q.Q. supervised the analysis. L.L. and X.H.H. framed the results and drafted the manuscript. K.R.B. and W.T. made revisions on the draft and approved the final version. Z.Q.Q. supervised the whole study process and is guarantor.

## Supporting information

Supporting information
