# Supplementary material for: The association between hyperuricemia and coronary artery calcification development: A systematic review and meta‐analysis
Source: Clin Cardiol. 2019 Sep 30;42(11):1079–86. doi: 10.1002/clc.23266 (PMC6837029; doi:10.1002/clc.23266)
Supplement: Supplementary file 1 — Supporting information [file CLC-42-1079-s001.docx]

| MeSH term search | Keyword search |
| --- | --- |
| 1.Uric acid | 3.urate |
| 2.hyperuricemia | 4.coronary artery calcification |
|  | 5.coronary calcification |
|  | 6.coronary artery calcium score |
|  | 7.coronary calcium |
|  | 8.coronary artery calcium scoring |
|  | 9.Coronary calcium |
|  | 10.Coronary calcium score |
|  | 11.Coronary calcium scoring |
|  | 12.coronary artery calcinosis |
|  | 13.coronary calcinosis |
|  | 14.calcification of Coronary artery |
|  | 15.coronary artery calcium |
| 16. “1 OR 2 OR 3” | |
| 17. “4 OR 5 OR 6 OR 7 OR 8 OR 9 OR 10 OR 11 OR 12 OR 13 OR 14 OR 15” | |
| 18. “ 16 AND 17” | |
